# Diacerein mitigates endocrine and cardio-metabolic disruptions in experimental PCOS mice model by modulating AdipoR1/ PON 1

**DOI:** 10.1186/s12902-024-01639-9

**Published:** 2024-07-10

**Authors:** Mohd Zahoor ul haq Shah, Vinoy Kumar Shrivastava, Showkeen Muzamil, Kehinde S. Olaniyi

**Affiliations:** 1 Laboratory of Endocrinology, Department of Bioscience, Barkatullah University, Madhya Predesh, Bhopal, 462026 India; 2 Department of obstetrics and Gynecology, Centre for Reproductive Medicine, the Fourth Affiliated Hospital of school of Medicine, International Institutes of Medicine, Zhejiang University, Yiwu, 322000 China; 3 Molecular Biology Laboratory, Faculty of Veterinary Sciences and Animal Husbandary, SKAUST-K, Srinagar, India; 4 Department of Physiology, College of Medicine and Health Sciences, Cardio/Endo-metabolic and Microbiome Research Unit, Afe Babalola University, Ado-Ekiti, 360101 Nigeria

**Keywords:** DiacereinDiacerein, Polycystic ovarian syndrome, Cardiovascular, Oxidative stress, Insulin resistance

## Abstract

**Background:**

This study aimed to explore the impact of Diacerein (DIC) on endocrine and cardio-metabolic changes in polycystic ovarian syndrome (PCOS) mouse model.

**Methods:**

A total of 18 adult female mice (Parkes strain), aged 4–5 weeks, were randomly assigned to three groups, each comprising 6 animals, as follows: Group I (control), received normal diet and normal saline as vehicle for 51 days; Group II received Letrozole (LET; 6 mg/kg bw) for 21 days to induce PCOS; Group III received LET, followed by daily oral gavage administration of DIC (35 mg/kg bw) for 30 days.

**Results:**

This study indicates that treatment with LET resulted in PCOS with characteristics such as polycystic ovaries, elevated testosterone, weight gain, visceral adiposity, high levels of insulin as well as fasting blood glucose in addition to insulin resistance, improper handling of ovarian lipids, atherogenic dyslipidemia, impaired Na + /K + -ATPase activity and serum, cardiac, and ovarian oxidative stress. Serum/ovarian adiponectin levels were lowered in LET-treated mice. In mice treated with LET, we also discovered a reduction in cardiac and serum paraoxonase 1 (PON1). Interestingly, DIC restored ovarian andcardio-metabolic abnormalities in LET-induced PCOS mice. DIC prevented the endocrine and cardio-metabolic changes brought on by letrozole-induced PCOS in mice.

**Conclusion:**

The ameliorative effects of DIC on letrozole-induced PCOS with concurrent oxidative stress, abdominal fat deposition, cardiac and ovarian substrate mishandling, glucometabolic dysfunction, and adiponectin/PON1 activation support the idea that DIC perhaps, restore compromised endocrine and cardio-metabolic regulators in PCOS.

## Introduction

Polycystic ovarian syndrome (PCOS) is a common endocrinological and reproductive condition characterized by hyperandrogenism, polycystic ovaries, and ovulatory dysfunction, affecting 6%–10% of females. PCOS is linked to metabolic issues such atherosclerosis, insulin resistance, obesity, dyslipidemia, and heightened cardiovascular disease risk [1]. In women with PCOS, endocrine disruption is linked to metabolic and reproductive abnormalities [2]. The primary metabolic abnormalities in PCOS are excess adiposity and disturbed lipid and glucose metabolism [3–6]. Changes in lipid-regulating genes are associated with hyperandrogenism, insulin resistance, infertility, and oxidative stress in PCOS [7]. The secretion of androgen by the ovary and adrenal glands is also increased by adiposity in PCOS.

In women with PCOS, the lipid and glucose regulating enzyme paraoxonase 1 (PON1) has already been linked to the emergence of cardio-metabolic abnormalities [8, 9]. PON1 may contribute to high density lipoprotein (HDL)'s antiatherogenic, anti-inflammatory, and antioxidant properties [10, 11]. PON1 also affects the modulation of glucometabolism, including fasting blood glucose, insulin sensitivity, glucose tolerance, and an upsurge in the expression of muscular glucose receptor 4 [12]. Consequently, PON1 may offer protection against conditions like diabetes, metabolic syndrome, cardiovascular disease, and PCOS that have decreased insulin levels [12]. It is worth noting that in PCOS females, diminished PON1 activity is connected to dyslipidemia, elevated circulating testosterone, insulin resistance, endothelial dysfunction, inflammation, and oxidative stress, all of which are significant factors that contribute to cardiovascular disease [13, 14].

The adipose tissue possesses the ability to produce adipocytokines, such as adiponectin, which exhibits various effects on glucose metabolism, lipid regulation, antioxidant functions, and inflammation reduction. This endocrine organ contributes to the regulation of metabolic processes [15]. The decline in adiponectin release observed in individuals with PCOS suggests a connection between adipose tissue overgrowth and dysfunction in PCOS development [8]. Furthermore, reports have it that increased paternal adiponectin transcription offers protection against adipose tissue dysfunction [16]. The receptors responsible for regulating adiponectin's actions are widely distributed throughout the hypothalamic-pituitary-ovarian axis and reproductive system, enabling the hormone to exert its effects on steroidogenesis and metabolism in these tissues [17, 18].

Letrozole, an aromatase inhibitor, causes PCOS in female mice within three weeks after starting treatment by raising testosterone production via inhibition of aromataze activity. Because it resembles human PCOS with equivalent metabolic, endocrine, and reproductive abnormalities like insulin resistance, impaired glucose tolerance, visceral adiposity, elevated circulating androgen and luteinizing hormone, polycystic ovaries, reduced follicle-stimulating hormone, etc., the letrozole-induced PCOS mice model offers a special chance to study human PCOS [19].

Since the pathophysiology of PCOS is complicated, it has not yet been fully understood. Additionally, it also takes into account whether genetics, dietary habits, and environmental elements all play a role [20]. PCOS is currently managed with a range of medications, such as metformin and clomiphene citrate. Unfortunately, these medicines have been related to negative side effects, including arthritis, lactic acidosis, toxicity to the liver, renal insufficiency, irregular bleeding, and stress [21–23]. Therefore, an acceptable and effective curative treatment has always been required from a medical point of view.Diacerein. Diacerein (DIC), is an anthraquinone component that has been proven effective for the treatment of osteoarthritis [24], and elicits an extensive range of antioxidant, anti-apoptotic, and anti-inflammatory actions according to past studies [25–27]. Some clinical studies [28, 29] have shown that DIC has positive effects on insulin secretion, hyperandrogenism, and metabolic parameters. It has been discovered that IL-1 serum levels in PCOS patients, whether obese and non-obese, are abnormally elevated [30]. Moreover, such PCOS female’s infertility is strongly associated with IL-1 [31]. In light of all of these data, it is clear that DIC may be effective in the management of PCOS. However, research using DIC as a treatment, mainly in PCOS-associated metabolic and reproductive dysregulation, are uncommon in rodents or humans. As a result, we expected DIC would improve PCOS-associated endocrine and cardio-metabolic derangements via adiponectin and PON1 modulation.

## Materials and methods

### Animals and treatment

All experimental procedures and reporting adhered to the ARRIVE guidelines 2.0. A standard set of environmental conditions was maintained, including a light–dark cycle of 12 h (06:00–18:00), unrestricted access to tap water and food, a temperature range of 23–26 °C, and relative humidity between 50–60%. Eighteen adult female mice (Parkes strain; aged 4–5 weeks) weighing 18–21 g were procured from Jeeva life sciences, Hyderabad, India, and were fed a standard chow diet. After one-week of acclimation, the mice were randomly assigned into three groups, each consisting of six mice. Group 1 (control) received normal saline as vehicle (0.9%) and standard chow for a duration of 51 days. Group II received Letrozole (LET; 6 mg/kg, oral gavage) dissolved in normal saline water (0.9%) for 21 days to induce PCOS, followed by a 30-day period without treatment. Group III received LET (6 mg/kg) for 21 days. Thereafter, the mice were treated with DIC (35 mg/kg bw) for 30 days. The final body weights of the mice were determined.

### Sample collection

The mice were anasthetized at the conclusion of the procedure with sodium pentobarbital (50 mg/kg; *ip*). Cardiac incision was used to take blood, which was later centrifuged for five minutes at 3000 rpm inside a plain tube. The serum was kept refrigerated till it was required for the biochemical analysis. In order to avoid any potential body weight-related fluctuation, the heart, ovaries, were removed, freed of any clinging connective tissues, wiped, and weighted. A portion of each organ was removed carefully just after ovary and heart was weighed. Then it was homogenized using a glass homogenizer in buffer phosphate and centrifuged at 8000 g for 10 min at 4 °C.

### Visceral adiposity

After the collection of blood samples, mice were dissected and all intra-abdominal fat deposits, which included the urogenital, were separated, quantified, and adjusted to body mass to reduce variability.

### Glucometabolic regulation

Just before mice were sacrificed, the fasting blood glucose (FBG) was determined. For this examination, all mice were fasted for 12 h and then blood was taken out from tail vein. The FBG levels were colorimetrically measured using a kit from Elabscience Biotechnology, Wuhan, Hubei, China Homeostatic model assessment of insulin resistance (HOMA-IR) was used to evaluated IR = fasting glucose (mmol/l) * fasting insulin (U/l)/22.5 [32].

### Biochemical analysis

#### Serum hormonal analysis

Concentrations of testosterone, estrogen, luteinizing hormone (LH)/follicle stimulating hormone (FSH) were measured using ELISA kits obtained from ELK biotechnology (Wuhan, China). The ELISA kits utilized the competitive inhibition enzyme immunoassay methodology. Each kit contained a microplate well (MW) pre-coated with a specific protein. Biotin-conjugated antibodies targeting LH, testosterone, estrogen, FSH, and progesterone were added to their respective MWs along with standards or samples. Avdin-horseradish peroxidase (HRP) was then added to each MW, followed by the addition of a TMB substrate solution and subsequent incubation. The resulting color shift, indicative of the enzyme–substrate reaction, was measured at a wavelength of 450 ± 10 nm using an ELISA reader. The reaction was stopped using the stop solution provided in the kit.

#### Serum insulin

Serum insulin content was measured using ELISA kits obtained from Elabscience Biotechnology (Wuhan, Hubei, China), following the instructions provided by the manufacturer.

#### Serum and ovarian adiponectin

Using kits purchased from Elabscience Biotechnology Wuhan, Hubei, China the quantities of serum and ovarian adiponectin was measured.

#### Serum and cardiac PON1 concentrations

Using kits purchased from Elabscience Biotechnology (Wuhan, Hubei, China) the quantities of serum and cardiac PON1 were measured in the serum and homogenates of ovarian and cardiac tissues.

#### Lipid profile

Using established colorimetric techniques and reagents from Meril Diagnostics (Gujrat, India) triglyceride (TG), total cholesterol (TC), and high-density lipoprotein-cholesterol (HDL-C) were determined in the serum, heart, and ovarian tissue homogenates. Additionally, the equation of Friedwaldwas employed to compute the levels of serum, cardiac, and ovarian LDL and VLDL (atherogenic lipid indicators).

### Lipid peroxidation and tissue injury biomarkers

#### Malondialdehyde (MDA) is a marker of lipid peroxidation

Malondialdehyde was determined in serum heart, and ovarian tissue using a traditional non-enzymatic spectrophotometric method and testing kits from Randox Laboratory Ltd. (U.K.). This method produces an MDA-TBA dimer that may be detected spectrophotometrically by reacting the sample's MDA with thiobarbituric acid (TBA).

#### Superoxide dismutase

The superoxide dismutase (SOD) activity was evaluated by taking pyrogallol of 100 ml and 2.9 ml of tissue homogenate supernatant (10%). SOD activity was expressed as units per gram of moist tissue.

#### Lactate and lactate dehydrogenase concentration

Serum, heart, and ovarian tissue supernatants were examined for lactate concentration and activities of lactate dehydrogenase (LDH) utilizing approved non-enzymatic and enzymatic colorimetric techniques using an assay kit purchased through Randox Laboratory Ltd.

#### Analysis of Ca2 + , Na + /K + , and H ^+^ ATPase activity in the ovarian and cardiac tissues

In the test tube, the following substances were added in volumes of 0.5 ml each: 0.35 M sodium chloride and 1.75 mM potassium chloride for Na + /K + ATPase. The mixture was then incubated at 37 °C for 60 min, and the reaction was stopped by adding 0.8 ml of ice-cold 10% (w/v) trichloroacetic acid (TCA). Centrifugation was performed at 4 °C and 4000 rpm for 20 min. Afterward, ascorbic acid (1 ml of 25%) was added to 1 ml of the supernatant, followed by a 20-min incubation at room temperature. The absorbance of Na + /K + was measured at 725 nm using a spectrophotometer, following previously described techniques [33]. Enzyme evaluation was conducted using the Evans method [34]. A waiting period of 10 min preceded the addition of ammonium molybdate (1 ml of 1.25%) to 1 ml of the supernatant to activate H + ATPase. Similarly, after the addition of ascorbic acid (1 ml of 9%), enzyme activity was evaluated using the Evans method. The absorbance at 725 nm was determined using a spectrophotometer [34]. Enzyme activity per mg of protein per hour was calculated based on the usage of 10–3 mol of pi.

#### Reverse transcription and real-time PCR

Total RNA extraction was performed using the TRI Reagent (Invitrogen) following the supplier's instructions. Subsequently, cDNA was synthesized from 1 µg of total RNA using the cDNA Synthesis Kit (Invitrogen). RT-PCR was conducted using SYBR green and real-time PCR methods. The mRNA values were determined from the standard curve based on the expression levels of each gene. For reference, the primers used in this study can be found in Table 1 in the supplementary materials. As an internal check, actin was utilized (Table 1).

**Table 1:** Primers employed for the analysis of RT-PCR

**Gene**	**Forward primer (5ˈ –3ˈ)**	**Reverse primer (3ˈ –5ˈ)**
**AdipoR1**	CGC TTT CTG CGT ATC GTC TG	CCA ACC TGC ACA AGT TCC CTT
**Actin**	TACGTCGCCCTGGATTTT	ATGAAAGAGGGCTGGAAGAG

### Evaluation of histology of ovaries

Ovarian tissue was fixated in 10% formol-saline for 24 h, dried, covered with paraffin, and sections were cut at a thickness of 5 m using hematoxylin & eosin (H & E) stains. Motic microscope was used to produce and evaluate the slides.

### Data analysis and statistics

Every set of data were presented as means ± SEM. Graph pad prism 8 was employed to conduct the statistical group analysis. One-way analysis of variance (ANOVA) was used to compare the mean values among the groups. The importance of pair-wise contrasts of average value amongt groups was determined using Tukey's test. At p value less than 0.01 statistical significance was accepted.

## Results

### DIC decreases body weight and adiposity in PCOS mice

**Fig. 1:**
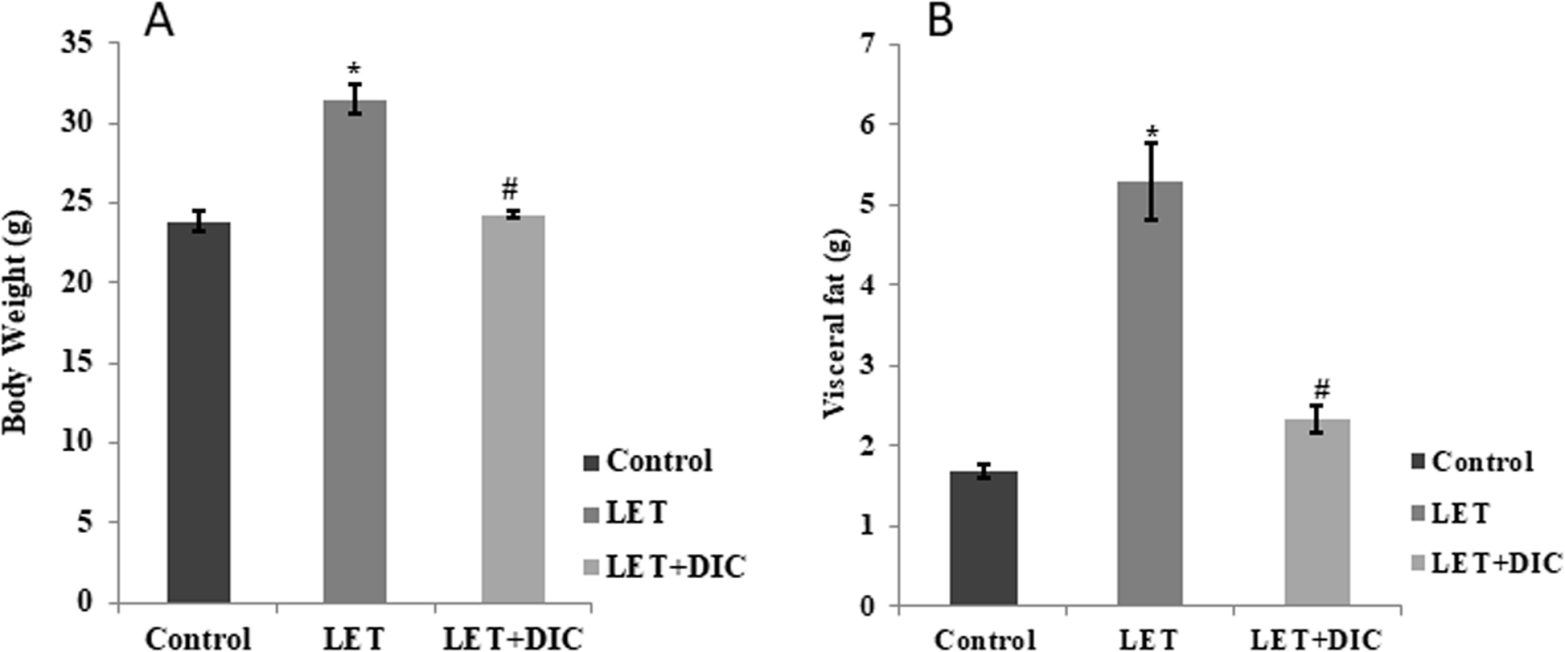
Diacerein decreased reversed body weight gain (**A**), and visceral fat (**B**) in LET-induced PCOS mice. Control: saline solution at 0.9%: LET (6 mg/kg of LET); LET + DIC (6 mg/kg of LETZ and 35 mg/kg of DIC); *P = 0.01 Control versus LET; #*P* = 0.01 LET versus LET + DIC. LET: Letrozole, DIC: Diacerein

The LET-treated group showed increased (*P* < 0.01) body weight in contrast to the normal control group, but the LET + DIC group decreased (*P* < 0.01) excess body mass in comparison to the LET-treated group. Visceral obesity is increased in the LET-induced PCOS mouse model, but was reduced by LET + DIC treatment (Fig. 1).

### DIC corrects glucometabolic imbalance in mice with PCOS

**Fig. 2:**
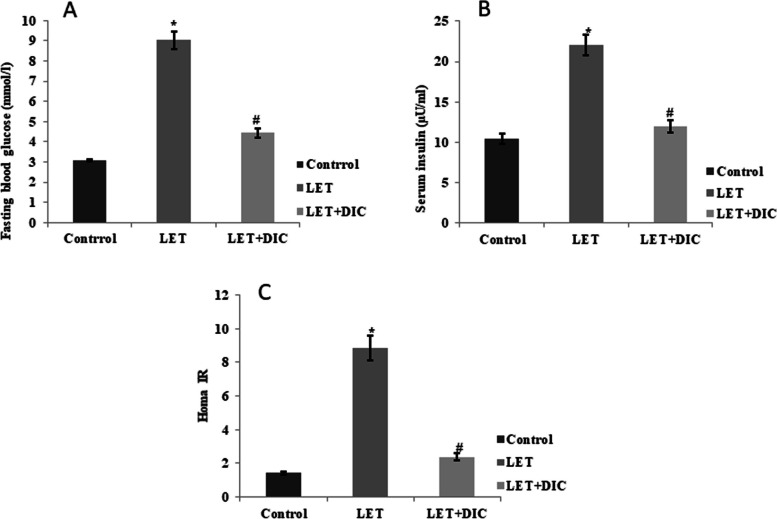
Diacerein decreased fasting glycemia (**A**), insulin (**B**) and HOMA-IR (**C**) in LET-induced PCOS mice. Control: saline solution at 0.9%: LET (6 mg/kg of LET); LET + DIC (6 mg/kg of LETZ and 35 mg/kg of DIC); *P = 0.01 Control versus LET; #P = 0.01 LET versus LET + DIC. LET: Letrozole, DIC: Diacerein

Compared with the normal control, letrozole treatment caused an increase *(P* < 0.01) in fasting blood sugar, however LET + DIC reduced (*P* < 0.01) it in comparison with LET-treated group. The elevated HOMA-IR (*P* < 0.01) in LET-treated group compared with control group demonstrates that they have insulin resistance. However, in comparison to the LET-treated group, the LET + DIC treatment resulted in better insulin sensitivity as evidenced by a drop in HOMA-IR score (*P* < 0.01). In comparison to the control group, the PCOS mice showed hyperinsulinemia; however, when compared to the untreated PCOS mice, DIC mitigated this hyperinsulinemia (*P* < 0.01) (Fig. 2).

### DIC improves hormonal imbalance in PCOS mice

**Fig. 3:**
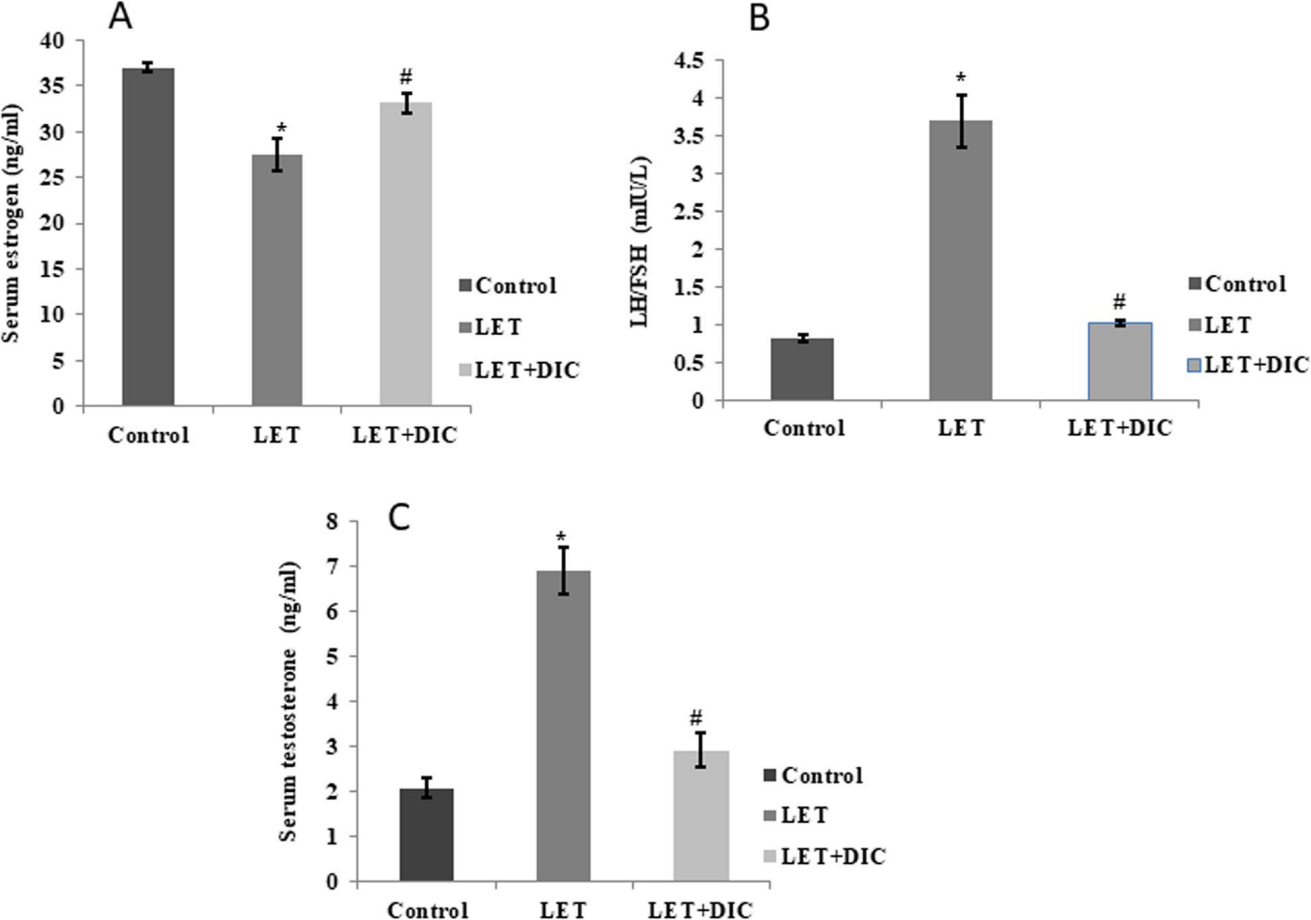
Diacerein increased serum estrogen (**A**) and LH/FSH (**B**) with a decrease in testosterone (**C**) in LET-induced PCOS mice. Control: saline solution at 0.9%: LET (6 mg/kg of LET); LET + DIC (6 mg/kg of LETZ and 35 mg/kg of DIC); *P = 0.01 Control versus LET; #*P* = 0.01 LET versus LET + DIC. LET: Letrozole, DIC: Diacerein

Compared with the standard control, the PCOS mice showed higher (*P* < 0.01) serum levels of LH and testosterone, while having lower (*P* < 0.01) levels of estrogen. In comparison to the LET-treated group, LET + DIC treatment resulted in a decreased (*P* < 0.01) e serum levels of LH and testosterone. Conversely, in comparison to LET-treated group, estrogen levels were significantly increased (*P* < 0.01) by the LET + DIC treatment (Fig. 3). Additionally, LET treatment caused an increase in the LH:FSH ratio, which was declined (*P* < 0.01) in the group treated with LET + DIC (Fig. 3).

### DIC improves serum, cardiac and ovarian lipid metabolism in mice with PCOS

**Fig. 4:**
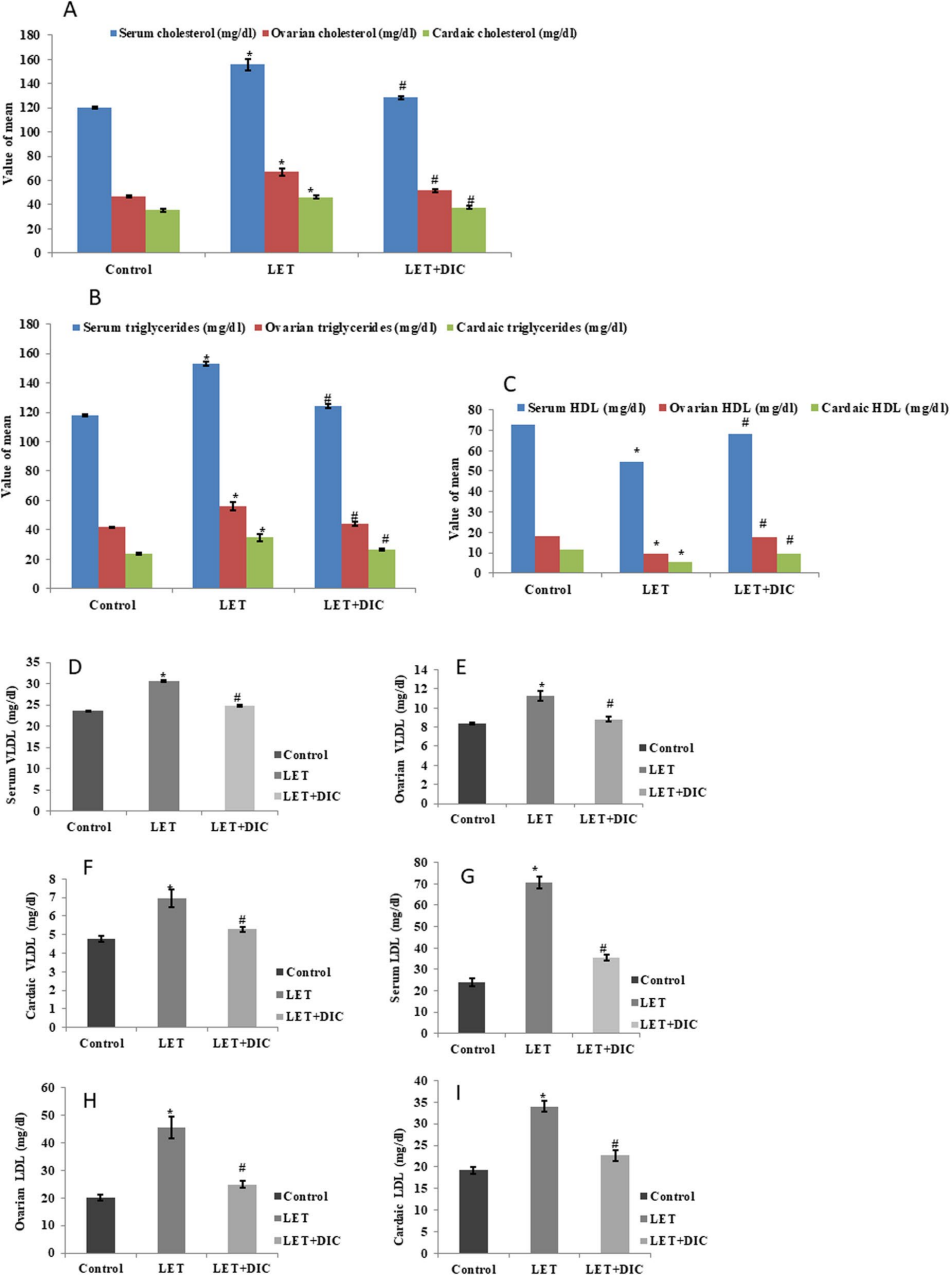
Diacerein decreased serum,cardiac and ovarian cholesterol (**A**), TG (**B**),VLDL (**D**-**F**), LDL (**G**-**I**) with the increase in HDL (**C**) in LET-induced PCOS mice.Control: saline solution at 0.9%: LET (6 mg/kg of LET); LET + DIC (6 mg/kg of LET and 35 mg/kg of DIC); *P = 0.01 Control versus LET; #P = 0.01 LET versus LET + DIC. LET: Letrozole, DIC: Diacerein, TG: Triglycerides

LET treatment caused a rise (*P* < 0.01) in total cholesterol/triglyceride with a decline in HDL concentrations in serum, ovarian and cardiac tissues in contrast to the standard control group. However, in comparison to the LET-treated group, LET + DIC treatment reduced (*P* < 0.01) total triglyceride/cholesterol levels along with an increase in HDL concentration in serum, heart, and ovarian tissues. Moreover, by comparing the LET-treated and control groups, LET treated elevated (*P* < 0.01) serum, cardiac, and ovarian indices of atherogenic dyslipidemia (LDL and VLDL), but LET + DIC treatment lowered (*P* < 0.01) these indices in the tissues and serum (Fig. 4).

### DIC suppresses LDH in serum and heart of mice with PCOS

**Fig. 5:**
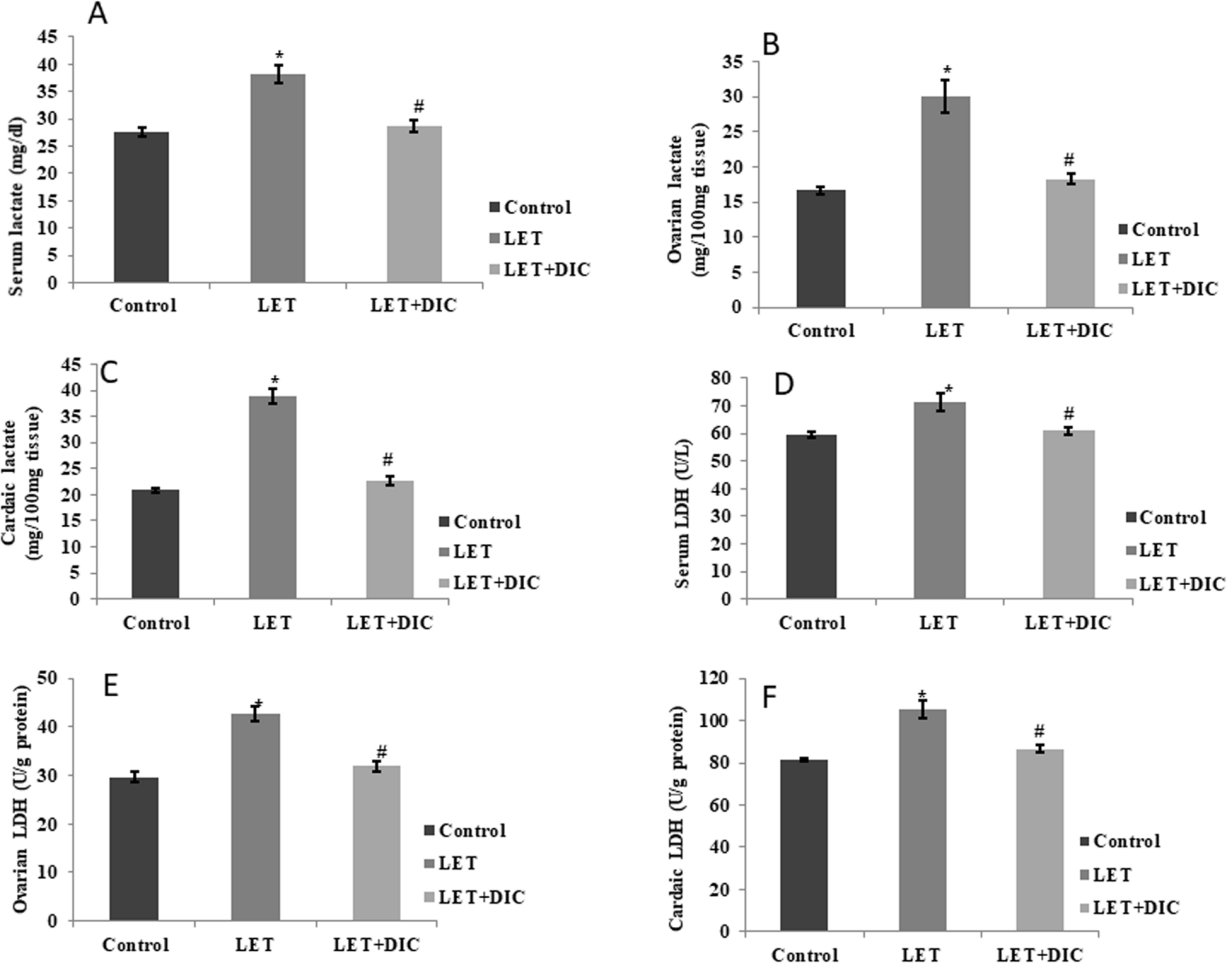
Diacerein decreased serum, cardiac and ovarian lactate (**A**-**C**) and lactate dehydrogenase (**D**-**F**) in LET-induced PCOS mice. Control: saline solution at 0.9%: LET (6 mg/kg of LET); LET + DIC (6 mg/kg of LET and 35 mg/kg of DIC); *P = 0.01 Control versus LET; #*P* = 0.01 LET versus LET + DIC. LET: Letrozole, DIC: Diacerein

The levels of lactate were increased in serum as well as ovarian tissue of LET-induced PCOS mice in comparison with control group. However, LET + DIC treatment caused a decline (*P* < 0.01) in the serum/ovarian lactate concentrations in comparison to the LET-treated group. Furthermore, LET treatment increased (*P* < 0.01) the activity of LDH in comparison to the control, which was suppressed significantly (*P* < 0.01) in the LET + DIC treated group (Fig. 5).

### DIC reduces oxidative damage in the heart and ovaries of mice with PCOS

**Fig. 6:**
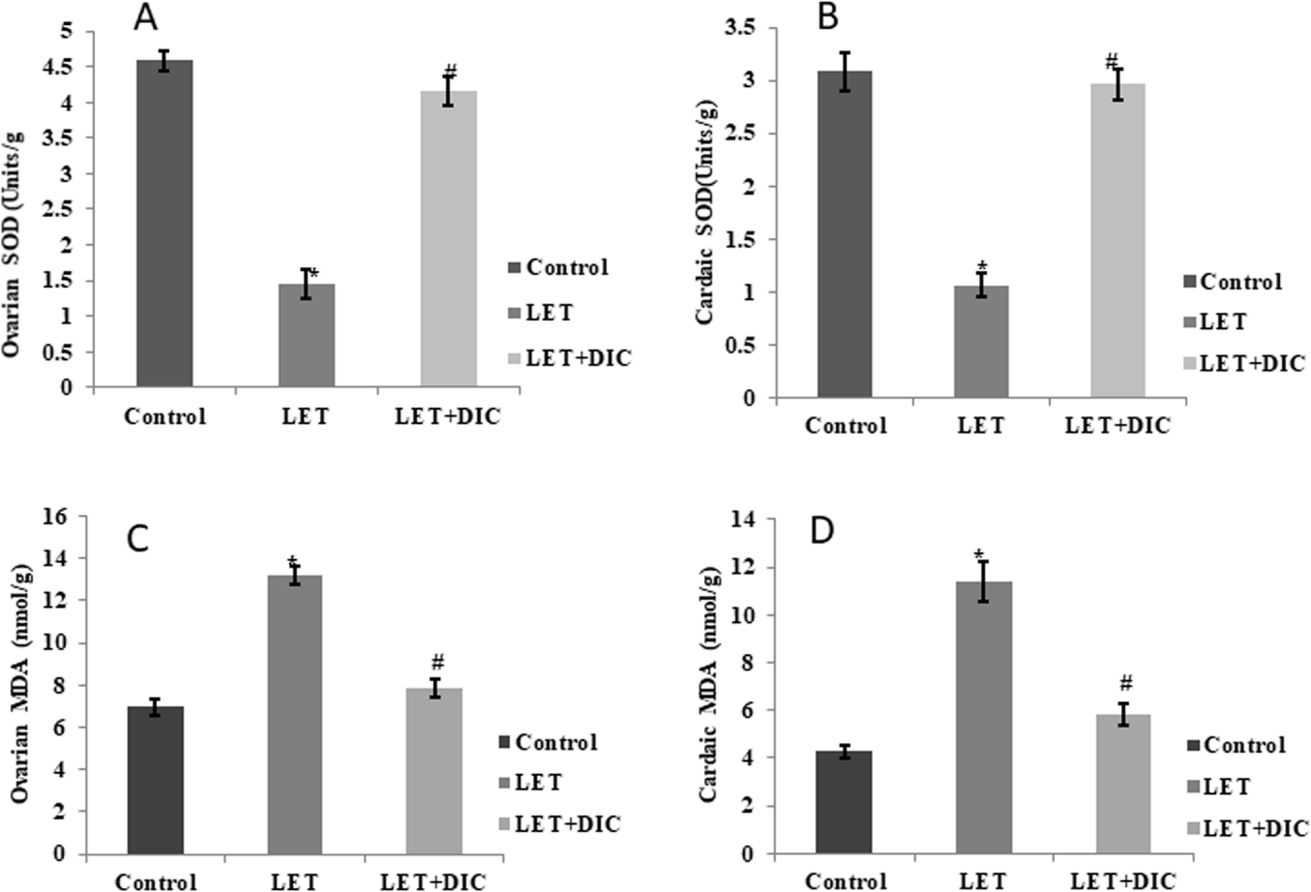
Diacerein increased ovarian and cardiac SOD (**A**-**B**) with the decrease in MDA (**C**-**D**) in LET-induced PCOS mice. Control: saline solution at 0.9%: LET (6 mg/kg of LET); LET + DIC (6 mg/kg of LET and 35 mg/kg of DIC); *P = 0.01 Control versus LET; #*P* = 0.01 LET versus LET + DIC. LET: Letrozole, DIC: Diacerein

Ovarian and cardiac malondialdehyde (MDA) concentrations were raised, while the SOD concentrations were declined by LET treatment in comparison to control. However, LET + DIC treatment decreased the concentration of MDA in ovarian and cardiac tissue in comparison to the LET-treated group. Moreover, LET + DIC increased (*P* < 0.01) SOD concentrations when compared with LET-treated group (Fig. 6).

### DIC increases the activity of ATPase (Na + / K + , H + ATPase) in ovary and heart of mice with PCOS

**Fig. 7:**
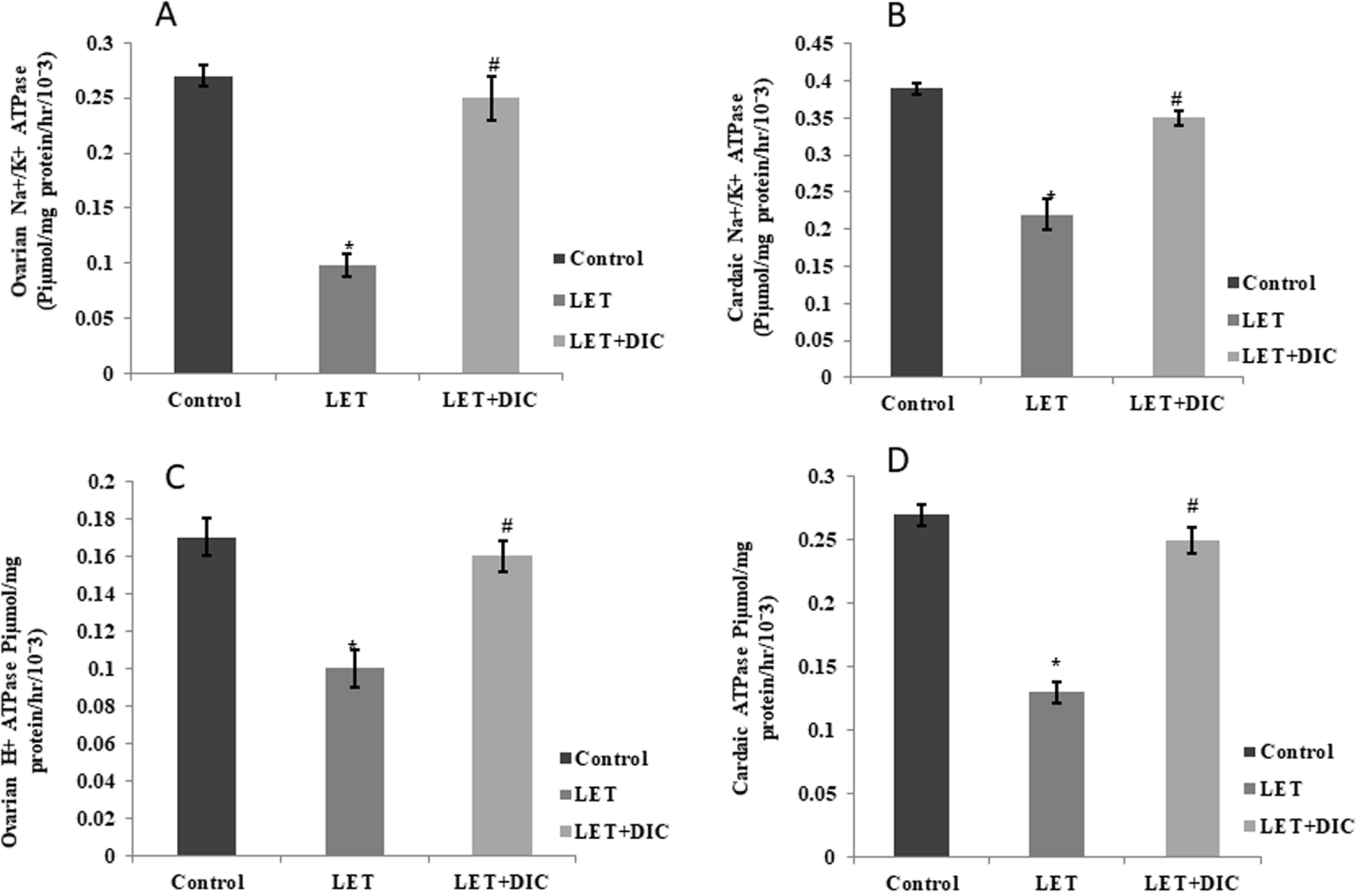
Diacerein increased ovarian and cardiac NA^+^K^+^ (**A**-**B**) and H^+^ ATPase (**C**-**D**) in LET-induced PCOS mice. Control: saline solution at 0.9%: LET (6 mg/kg of LET); LET + DIC (6 mg/kg of LET and 35 mg/kg of DIC); *P = 0.01 Control versus LET; #P = 0.01 LET versus LET + DIC. LET: Letrozole, DIC: Diacerein

LET treatment decreased (*p* < 0.001) the activities of the proton pump enzymes (Na + / K + and H + ATPase) in the heart and ovary of mice in comparison to the control. However, LET + DIC treatment caused an elevation (*P* < 0.01) in Na + / K + and H + ATPase activities in comparison to the LET-treated group (Fig. 7).

### DIC increased adiponectin concentration in both serum and ovary of mice with PCOS

**Fig. 8:**
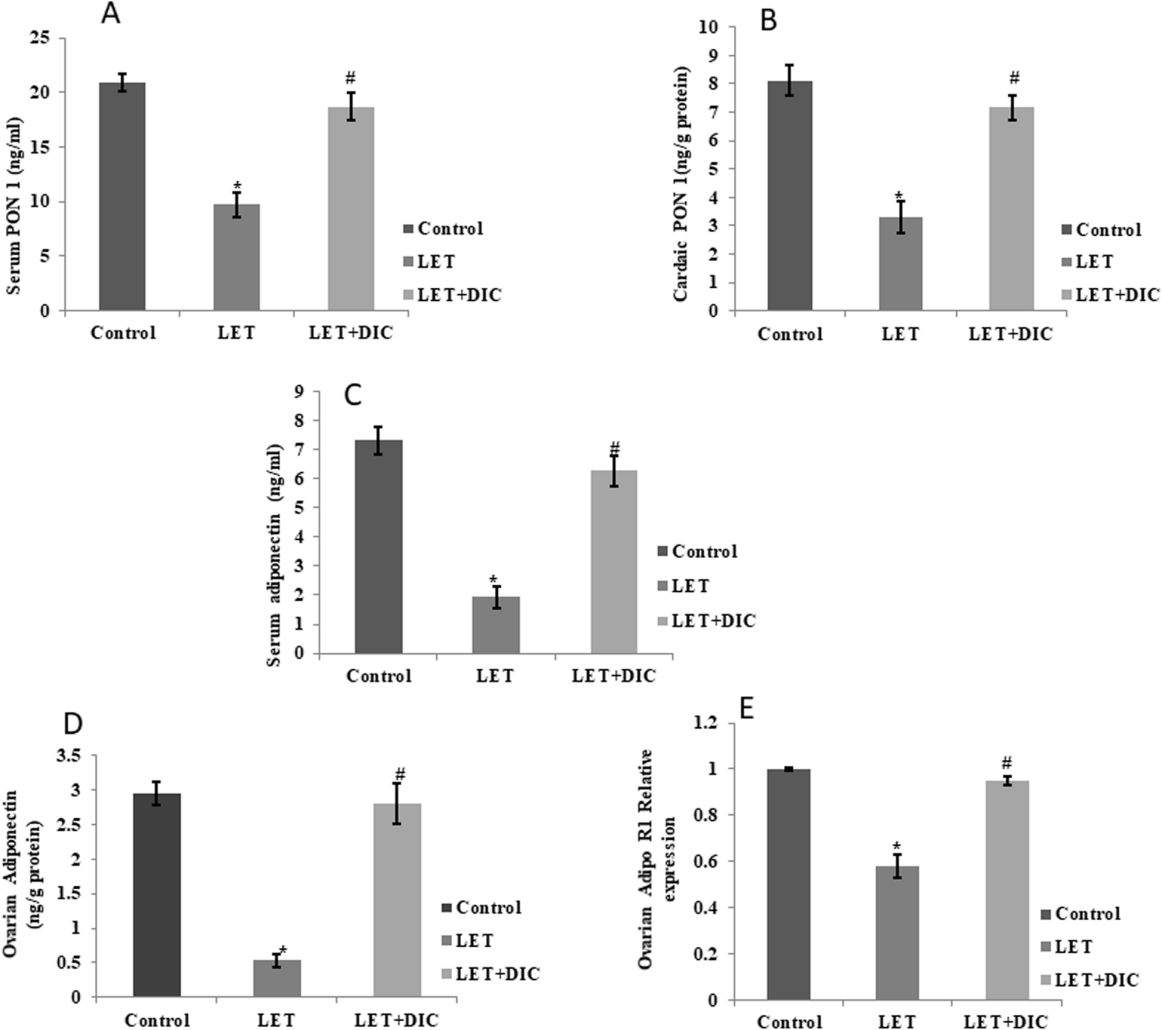
Diacerein increased serum and cardiac PON 1 (**A**-**B**), serum and ovarian adiponectin/Adipo R1 (**C**-**E**) in LET-induced PCOS mice. Control: saline solution at 0.9%: LET (6 mg/kg of LET); LET + DIC (6 mg/kg of LET and 35 mg/kg of DIC); *P = 0.01 Control versus LET; #*P* = 0.01 LET versus LET + DIC. LET: Letrozole, DIC: Diacerein

Adiponectin concentrations were declined (*P* < 0.01) in serum as well as ovarian tissue of LET-induced PCOS mice in comparison with control. However, LET + DIC treatment caused an elevation (*P* < 0.01) in the serum/ovarian adiponectin concentrations in comparison to the LET-treated group (Fig. 8).

### DIC increased PON1 concentration in both serum and heart of mice with PCOS

When compared with the control, the PON1 concentration was declined (*P* < 0.01) in the serum as well as heart tissue of LET-treated mice. However, LET + DIC treatment caused an elevation (*P* < 0.01) in the serum/cardiac PON1 concentrations in comparison to the LET-treated group (Fig. 8).

### DIC improves ovarian histoarchitecture in PCOS mice

**Fig. 9:**
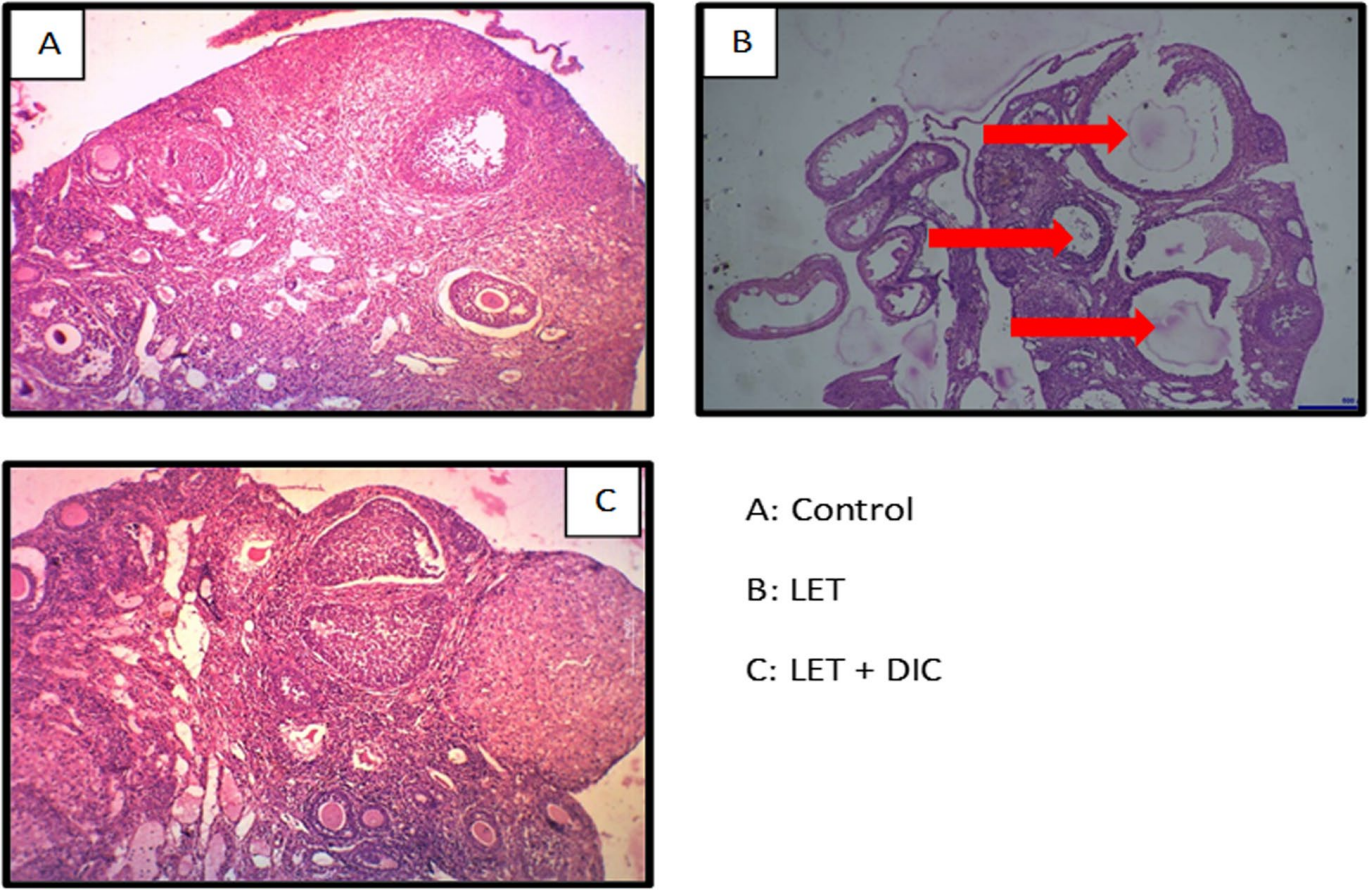
Diacerein attenuated ovarian cysts in LET-induced PCOS mice. Letrozole, DIC: Diacerein. Cystic follicles are identified with red arrows

The histopathological examination of a section of the ovary revealed distinct features of granulosa cells, secondary follicles, and oocytes. In comparison to the control group, mice with LET-induced PCOS exhibited signs of ovarian follicle degeneration, formation of follicular cysts, and distorted granulosa cells, with a notable absence of oocytes. In contrast, the ovarian tissue from the LET + DIC treated group displayed normal granulosa cells and an antral cavity with a clearly visible isolated oocyte (Fig. 9).

## Discussion

**Fig. 10:**
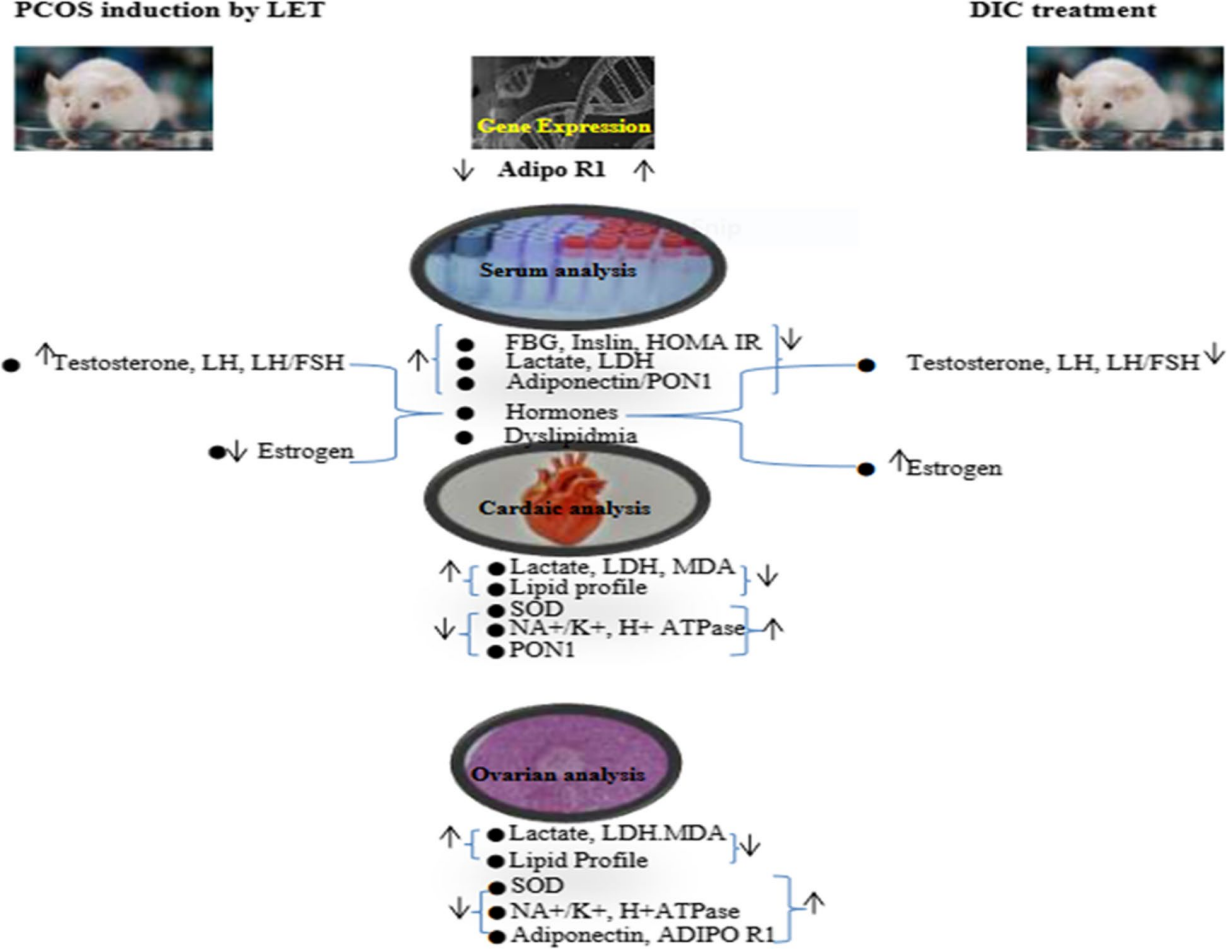
Schematic diagram showing the attenuation of endocrine and cardiometabolic abnormalities by DIC through Adipo R1/PON 1modulation in LET-induced PCOS mouse model Diacerein attenuated ovarian cysts in LET-induced PCOS mice. Letrozole, DIC: Diacerein

This study indicates that treatment with letrozole results in PCOS with characteristics that are similar to those of human PCOS, including polycystic ovaries, elevated serum testosterone, weight gain, visceral adiposity, high levels of insulin as well as FBG in addition to insulin resistance, improper handling of ovarian lipids, atherogenic dyslipidemia, impaired Na + /K + ATPase activity and serum, cardiac, and ovarian oxidative stress. Adiponectin levels in the serum/ovary were also lowered in LET-treated mice. In mice treated with LET, we also discovered a reduction in cardiac and serum PON1. As a result, LET-induced PCOS mimics the metabolic and reproductive abnormalities present in human PCOS. However, by upregulation of Adipo R1 and PON1, DIC reduced the endocrino-metabolic aberrations caused by letrozole in the serum, heart, and ovary (Fig. 10). Our decision to use DIC dosages of 35 mg/kg/day was made in light of both our preliminary findings and the effectiveness of these dosage in other research [35, 36].

Nevertheless, the present data demonstrate that DIC improves PCOS-associated cardio-metabolic derangements via adiponectin and PON1 modulation.

Many women within the age of reproduction are impacted by PCOS. Infertility means the inability of a sexually mature, non-contraceptive pair to conceive a child within a year after having unsafe sexual contact [37]. Around 20 and 35 percent of all cases of infertility are estimated, are influenced by female factors. These concerns are commonly a result of ovulatory issues, that are often defined by delayed or absent of menstrual cycles [38, 39]. Among the common signs of PCOS is obesity, which also includes excess weight, irregular periods, and infertility [40]. In additional to fertility problems, there are additional challenges like insulin resistance and cardiovascular disease (CVD). and abdominal obesity are additional clinical signs of PCOS that are linked to high testosterone levels and cause women to experience anxiety and depression [41].

Visceral fat deposition has indeed been related to insulin resistance, dyslipidemia, and increased CVD risks. For several PCOS patients, adiposity is likely primary source of insulin resistance [42]. Visceral fat gain has been linked to insulin resistance, dyslipidemia, as well increased CVD risks. In several Patients with PCOS, insulin resistance is likely caused by abdominal obesity [42]. The PCOS mouse model indicated increased body weight and excess abdominal fat, but the deficiencies were corrected by oral DIC treatment. In a latest study, letrozole-induced PCOS in mice resulted in elevated body weights [43].

Adipose In addition, etrozole treatment throughout the current study resulted in elevation of abdominal fat, diminished circulating and ovarian adiponectin concentration, insulin resistance, hyperinsulinemia, dyslipidemia, oxidative stress, and hyperandrogenemia, indicating an association with both adiponectin and PCOS metabolic changes. These findings are in accordance with previous studies on impaired adipose tissue activity in PCOS. Moreover, multiple studies have connected PCOS metabolic problems to decreased adiponectin levels. Adiponectin as well as its receptor Adipo R1 are widely distributed throughout reproductive tissues, especially the ovaries. This increased their capacity to affect steroidogenic processes and metabolic controls in the reproductive tissues [18, 44, 45]. The current study's findings that letrozole-induced PCOS mice have dyslipidemia, oxidative stress, impaired glucometabolic regulation, and decreased adiponectin concentration in serum and ovarian tissue are consistent with a previous study that revealed metabolic dysregulation in adiponectin-deficient mice [45]. Additionally, women with PCOS possess lower levels of adiponectin, which might also make them more susceptible to developing type 2diabetes due to decreased glucometabolic control and dyslipidemia, demonstrating a crucial metabolic role for adiponectin [46].

To assess the impact of DIC on hormonal changes, we conducted an analysis of serum hormone levels. Our findings revealed that DIC administration led to a decrease in serum testosterone, LH, and LH/FSH concentration in LET + DIC mice compared to those treated with LET alone. Elevated LH levels and an increased LH/FSH ratio have been identified as potential indicators of PCOS in females [47]. Moreover, in mice with PCOS, DIC treatment significantly lowered serum testosterone, LH, and LH/FSH levels. Reducing elevated testosterone levels can be beneficial for managing PCOS-related conditions, as high androgen levels have been implicated in the etiology of PCOS [48, 49]. In addition, LET-treated mice manifested decreased levels of serum estrogen, progesterone, and FSH. This decline corresponded to alterations in the growth and morphology of follicles in the ovary during mid or early-follicular stages [50]. Hormone-based therapies are commonly used to address atypical symptoms associated with female reproductive disorders. However, these treatments are often accompanied by side effects such as uterine hemorrhage and hyperplasia [51, 52]. The observed findings suggest that DIC may offer potential effectiveness in addressing hormonal imbalances associated with PCOS. Reduced steroid hormones levels in the ovary have been correlated with increased numbers and diverse shapes of developing follicles [53].

Oxidative stress (OS) is critical in the early phases of carcinoma conversions or cancer origination by boosting protooncogene and suppressing anticancer genes [54]. As a result, higher OS in PCOS may raise risk of developing cancer along with contributing to epigenetic changes. The most typical symptoms and suspected sources of ovulatory disruption in PCOS patients are inflammation, obesity, and hyperandrogenism, which have all been strongly linked to oxidative stress. Numerous studies have connected decreased proton (H +) pump (ATPase) action to issues with female reproductive. Some studies have suggested a relationship between oxidative stress and decreased ATPase activity [55]. Free radicals, inflammation, and membrane lipid peroxidation are all reported to significantly enhance the vulnerability of ATPase to these circumstances. This was discovered that lipid peroxidation (MDA) specifically altered the function of H + and Na + /K + ATPase [56]. Reactive species of oxygen might significantly increase quantities of helpful antioxidants, which could also assist in explaining why H + and Na + /K + ATPase function is hindered [57, 58]. In contrast to the LET-treated group, the group that received LET + DIC displayed a considerable increase in H + and Na + /K + ATPase.

PON1 is an anti-inflammatory and antioxidant agent which aids HDL in its function as a vasculo-protective agent and stops the generation of oxidized LDL [59]. Lowered PON1 activities in PCOS as a result of oxidative stress and lipid peroxidation contributed to the origin of PCOS issues [60]. PCOS decreases blood PON1 levels, which could be related to a higher risk of heart disease with atherosclerosis [10]. Additionally, it has been shown that in comparison to normal control patients, women with PCOS had lower serum PON1 activity [61]. Our study found that the letrozole-induced PCOS group had lower blood and ovarian PON1 levels than the control group, which was restored by DIC treatment, in agreement with other research. A possible explanation for the decreased PON1 activity is enhanced PON1 inactivation brought on by PCOS's elevated ROS generation [61]. As a result, by modifying the structure of the protein, superoxide anions might be accountable for PON1's reduced function. PON 1 is believed to be inactivated by ROS, perhaps as a result of xanthine oxidase producing superoxide anions [62]. It has recently been demonstrated that decreased PON1 activities enhances oxidative stress levels and alters metabolism in Patients with PCOS [62]. Therefore, the fact that LET-treated PCOS mice displayed higher levels of lipid peroxidation, lactate, and lactate dehydrogenase activities in their serum, hearts, and ovaries backs up the notion that oxidative stress plays a role in the development of PCOS. Additionally, it's possible that the oxidative stress in the LET-treated PCOS rats is related to the decreased serum and ovarian PON1 activity in these animals.

This study's key finding was that DIC restored LET-induced PCOS ovarian and cardio-metabolic abnormalities. In LET-induced PCOS mice, DIC treatment substantially increased energy metabolism, which restricted visceral fat gain, insulin resistance, improved hyperglycemia, decreased oxidative stress, and dyslipidemia. It also caused a reduction in circulating levels of testosterone, luteinizing hormone, and the LH/FSH ratio, along with improved hyperglycemia and decreased oxidative stress. Based on a histopathological evaluation, the administration of DIC improved the damaged cytoarchitecture of the ovaries brought on by LET treatment. Additionally, in LET-induced PCOS, DIC treatment resulted in decreased serum, cardiac, and ovarian lactate, LDH. Intriguingly, the beneficial effects of DIC treatment in LET-induced PCOS mice were accompanied by elevated levels of PON1 and adiponectin. Nevertheless, the study did not investigate the cause effect relationship between PON1/adiponectin and other biochemical changes, which for the basis for the future research.

## Conclusion

In summation, DIC administration prevented the endocrine and cardio-metabolic changes brought on by letrozole-induced PCOS in mice. As a result, the ameliorative effects of DIC on letrozole-induced PCOS with concurrent oxidative stress, abdominal fat deposition, cardiac and ovarian tissue mishandling, glucometabolic dysfunction, and Adiponectin/PON1 activation support the notion that DIC may be able to restore compromised endocrine and cardio-metabolic changes in PCOS. However, the future study would investigate the mechanisms underlying the modulation of adiponectin/PON1 by DIC in a mouse model of PCOS.

## Data Availability

The raw data will be made available from the corresponding author on request.
